# Rational use of Computed Tomography Scan head in the Emergency Department of a high volume tertiary care public sector hospital

**DOI:** 10.12669/pjms.35.2.719

**Published:** 2019

**Authors:** Tahira Nishtar, Tabish Ahmad, Nosheen Noor, Fayaz Muhammad

**Affiliations:** 1 *Dr. Tahira Nishtar, (FCPS), Associate Professor and Chairperson, Radiology Department, Medical Teaching Institute, Lady Reading Hospital, Peshawar, Pakistan*; 2 *Dr. Tabish Ahmad, PGR-FCPS II, Radiology Department, Medical Teaching Institute, Lady Reading Hospital, Peshawar, Pakistan*; 3 *Dr. Nosheen Noor, (MCPS, FCPS), Assistant Professor, Radiology Department, Medical Teaching Institute, Lady Reading Hospital, Peshawar, Pakistan*; 4 *Dr. Fayaz Muhammad, PGR-FCPS II, Radiology Department, Medical Teaching Institute, Lady Reading Hospital, Peshawar, Pakistan*

**Keywords:** CT scan, emergency department, rational use

## Abstract

**Objective:**

To emphasize the rational use of Computed Tomography (CT) head in emergency department (ED) of a high volume tertiary care hospital.

**Methods:**

This retrospective observational study was conducted in Radiology Department of Medical Teaching Institute Lady Reading Hospital (MTI-LRH), Peshawar, Pakistan from November 1^st^ 2017 to 31^st^ January 2018. Patients of all ages and both genders presenting to the emergency department with post traumatic and non-traumatic indications for emergency CT head scan were included in the study. The imaging was performed on GE 16 multi slice Optima CT system. The imaging protocol included slice thickness of 3-5mm, non-contrast study for cases of head trauma or suspected stroke. Where needed intravenous contrast was administered e.g. to exclude meningitis in patients presenting with severe headache. Patients undergoing CT examination for regions of the body other than head and brain were excluded from the study as their number was insignificant. Reporting was done on PACS and results analyzed using latest SPSS version.

**Results:**

Out of 4284 CT scans performed in emergency department 90.8% were CT head (3893). Among 3893 CT scan head done in ED, 2581 cases were reported normal (66.29%), while 1312 cases had positive findings (33.7%), including post traumatic and non-traumatic.

**Conclusion:**

Misuse of CT head is common especially in an emergency setting. Emergency physicians should be encouraged to obtain a detailed history and perform a thorough physical examination with reference to internationally standardized guidelines, while ordering CT scan.

## INTRODUCTION

CT scan is an important investigating tool in the ED in establishing a particular diagnosis or otherwise helping a physician to exclude one. So it is not only used as a screening tool but also as a diagnostic one.^1^

The most common CT scan done in an emergency department is CT head, which falls under two categories i.e. post traumatic and non-traumatic CT head.^2^ There are risks related to overuse of CT scans as each scan involves radiation, not justifying scans done for marginal reasons. The overuse of CT scan is closely related to changing trends in medical practice and easy access to CT scan with lower barriers and thresholds to performing the test.^3^

A segment of CT scan in emergency department is done for medico-legal purposes requiring evidence based clinical practice. There are obvious benefits to judicious imaging in ED with less ionizing radiation exposure. Radiation from medical imaging causes long term cancer risk more significantly in children and young adults. In the elderly and high risk population with co-morbidities, repeated CT head does not identify acute clinically significant findings.^4^

**Fig.1:**
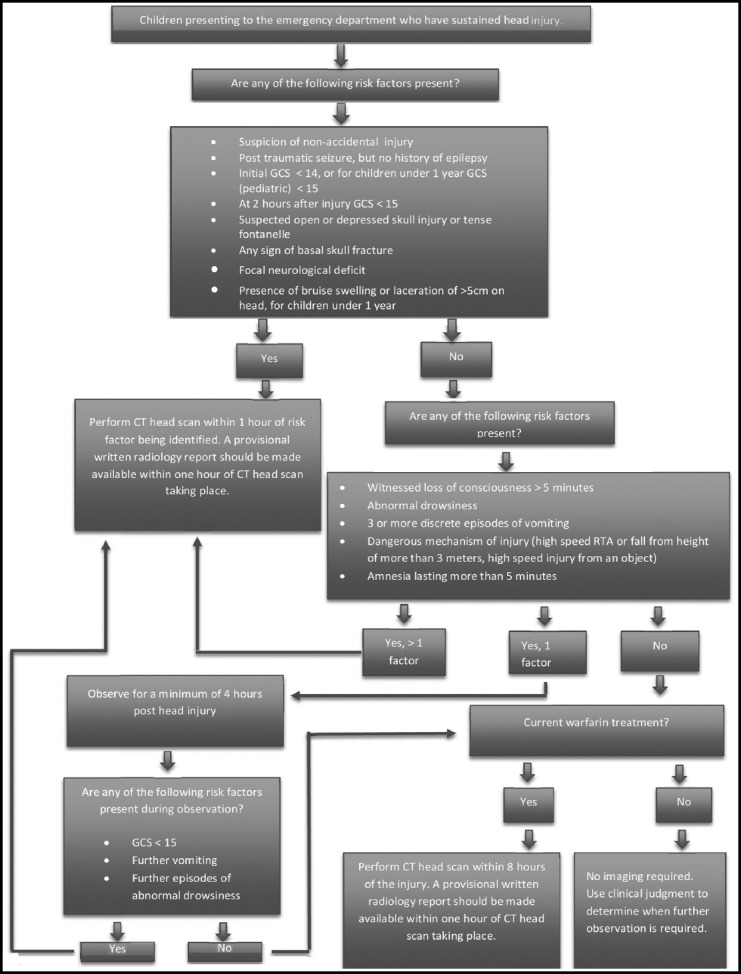
NICE guidelines for selection of children for CT head scan.^11^

There are certain pre-requisites for ordering a CT Scan of Head. It includes comprehensive history & physical examination and documentation concerning relevant symptoms related to ordering a specific CT examination with reference to the standardized international guidelines for CT imaging in an emergency setting (Fig.1, 2 & 3).

**Figure 2:**
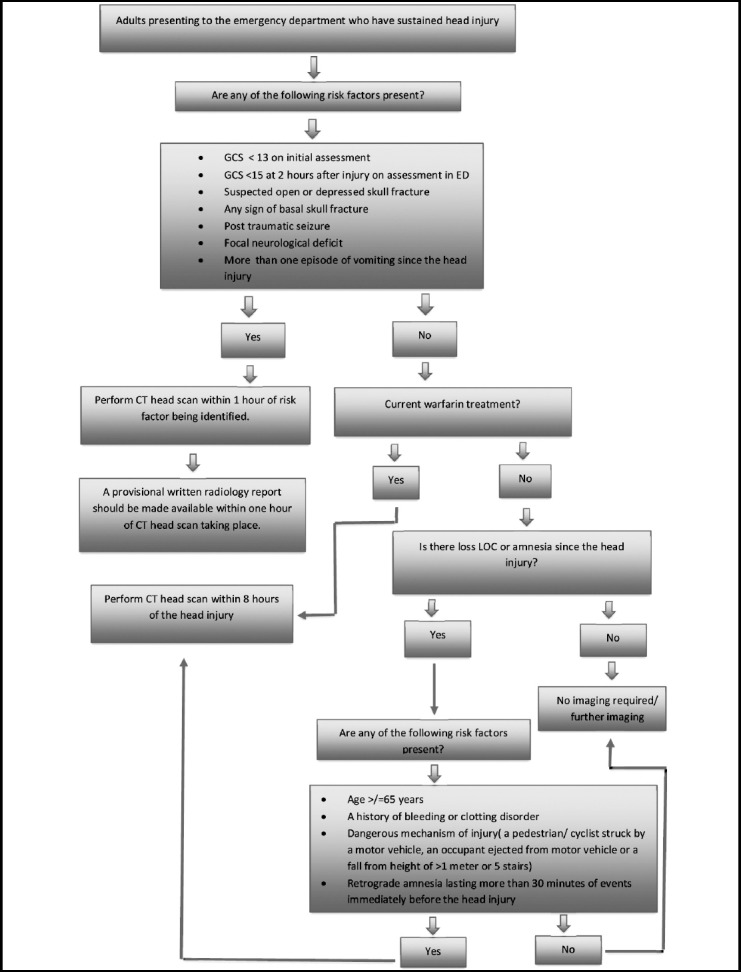
NICE guidelines for selection of adults for CT head scan.^11^

**Fig.3:**
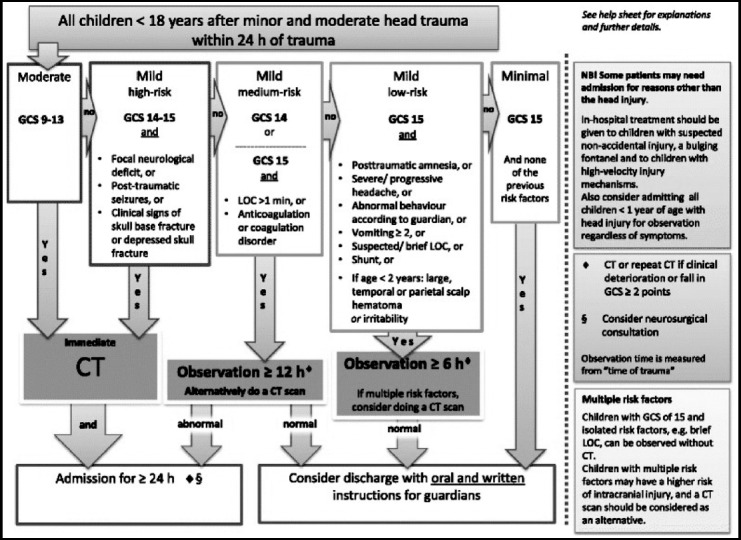
Scandinavian guidelines for initial management of minor and moderate head trauma in children.^12^

This is perhaps first study of its kind performed in a public sector hospital of the Pukhtunkhawa province of Pakistan. The purpose of this study was to emphasize on rational use of CT head in ED and to discourage misuse of CT scan in patient population.

## METHODS

This retrospective observational study was conducted in Radiology Department of Medical Teaching Institute Lady Reading Hospital (MTI-LRH), Peshawar, Pakistan from November 1^st^ 2017 to 31^st^ January 2018. Patients of all ages and both genders presenting to the emergency department with post traumatic and non-traumatic indications for CT head were included in the study. The imaging was performed on Optima 16 multi slice CT system (GE). The imaging protocol included slice thickness of 3-5mm, non-contrast study in case of trauma or stroke. Where needed intravenous contrast was administered e.g. to exclude meningitis. Patients undergoing CT examination for regions of the body other than head and brain were excluded from the study as their number was insignificant. In cases of stroke, finding of infarct rather than suspected bleed or vice versa were both considered as positive. Those patients who had normal CT were considered as negative. CT images were reported on PACS in two sessions of six hours each, with morning session reporting done by resident under the supervision of consultant radiologist while evening session reporting was performed by a senior Third or Fourth Year resident independently. Information was analyzed using latest SPSS version.

## RESULTS

Out of 4284 patients, 3019 were male (70.5%), while 1265 were female (29.5%). 4284 CT scans included 90.8% CT head (3893). The rest of 9.13% (391) cases included abdomino-pelvic, thoracic, and musculoskeletal. Among 3893 CT head scans done in ED, 2581 cases were reported normal, while 1312 cases had positive findings (Table-I). The cases with positive findings were further divided into two broad categories of indication; post-trauma and non-trauma cases (Table-II). Post traumatic cases included road traffic accidents (RTA), history of fall (HOF), fire arm injury (FAI) and physical assault, whereas non-trauma indications included stroke, unconsciousness, severe headache and clinically suspected meningitis or rarely to exclude space occupying lesion.

**Table-I:** CT Head (N=3893): Normal vs. Abnormal.

Indication	Normal CT head (2581)		Abnormal CT head (1312)	

	No.	Percentage out of total N (%)	No.	Percentage out of total N (%)
Non traumatic	1330	34.16	778	19.98
Post traumatic	1251	32.13	534	13.72

Total	2581	66.29	1312	33.7

**Table-II:** CT Brain with positive findings (N=1312).

Non trauma positive CT scans		Post trauma positive CT scans	
Intracranial hemorrhage	232	Subarachnoid hemorrhage	43
Cerebral infarcts	309	Subdural hemorrhage	46
Hydrocephalus	26	Extradural hemorrhage	54
Brain atrophy	211	Intraventricular hemorrhage	26
		Skull bone fractures	236
		Pneumocephalus	129

Total	778 (59.3%)	Total	534 (40.7%)

## DISCUSSION

Access to free technical facilities in emergency department (ED) spares the patients from queuing up to seek specialist appointments. This results in overcrowding and increasing number of patients seeking emergency care for non-urgent cases leading to “misuse of emergency department services”. The fact that emergency department offers a 24/7 free service adds to this overcrowding. Another factor contributing to emergency department overcrowding is in-adequate in-patient bed availability. Demand growth is mainly due to inaccessibility to primary healthcare.^1^

CT head is one of the most common scan prescribed by physicians in ED for various indications such as weakness, aphasia, headache, syncope, dizziness and trauma. However the number of CT scans ordered by the physicians varies from one hospital to another and from one physician to another because of lack of implementation of standardized protocols. The use of CT scans in the ED has significantly increased over the past decade. Some physicians rely more on their clinical history and examination findings while others on evidence of presence or absence of pathology in the form of a positive or negative CT scan.^5^ In this study conducted at ED, LRH, majority of the patients referred for CT head scan which were reported as normal presented with headache, history of seizures and minor trauma. In patients with minimal and mild head injury CT head was advised for screening. However, according to the international guidelines there is no need of undergoing an urgent CT head if GCS is 14 to 15 without risk factors, and either discharge or 6 to 12 hour observation is suggested accordingly, rather than performing screening CT head. The CT head scans with positive findings in our study were mainly post major trauma including RTA, falls, fire arm injuries and physical blow to the head. In cases of suspected stroke predominantly in elderly population, patients presenting with weakness, seizures, amnesia and loss of consciousness with or without focal neurological deficit underwent CT head. The most common finding in elderly was senile brain atrophy which was included in positive scans.

**Fig.4:**
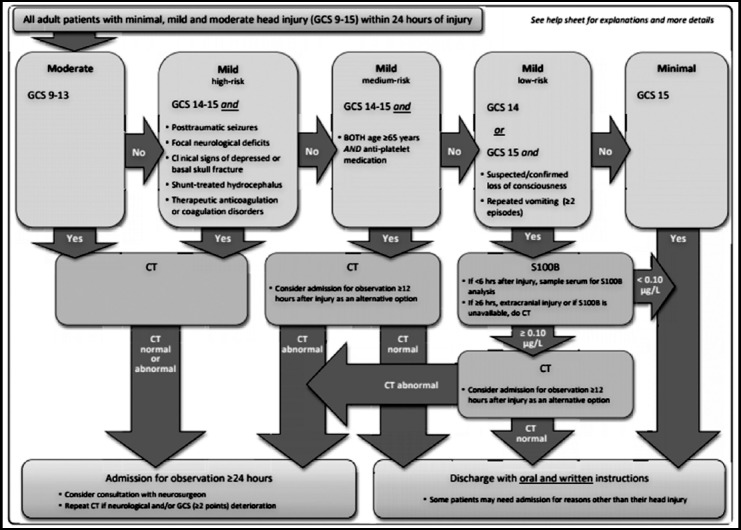
Scandinavian guidelines for initial management of minor and moderate head trauma in adults.^13^

It is very important to channelize the resources of hospital in an effective way in order to avoid not only unnecessary load on the ED CT services but also to decrease the radiation exposure to patients. Increased radiation exposure for patients visiting the ED has been a significant problem in recent years as the physicians working in ED have developed a low threshold for ordering such investigations.^1^ This trend has increased in the past few years.^6^ This increases the exposure of patients to unnecessary radiations; radiations that could have otherwise been avoided if the ED physician had taken a detailed history and performed a thorough physical examination with reference to the standardized guidelines before ordering a CT scan.^3^

The increased radiation exposure is associated with increased incidence of malignancies in the population.^7^ Children are particularly more sensitive to repeated radiation doses.^8^ On the other hand, elderly patients in the population are also at an increased risk particularly if there is gross abuse of emergency CT scan services.^7^,^8^

Over-ordering of unnecessary CT scans increases the duration of stay in ER which not only wastes the precious time of the health care providers working in the ED but also increases the exposure of such patients to various hospital acquired infections, which in most cases are drug resistant and hard to treat, thus increasing the overall morbidity and mortality in the population.^9^ Additionally it leads to overcrowding of the ED causing significant problems in delivery of quality services to the more deserving patients.^1^ One important reason for the overuse of CT services in the ED is the increased pressure on the ED physicians to make rapid and precise diagnosis in the shortest possible time. The pressure under which the ER physicians have to work forces them to order CT scans for increasing number of patients in order to screen them against common pathologies presenting to ED without taking a detailed history or performing a thorough physical examination.^2^ This indiscriminate use of CT scans in the ED has led to an enormous amount of financial burden on the hospital resources.^6^ The misuse of financial resources could otherwise prove very helpful if properly channelized to improve other services provided by the emergency department.^9^

In our study all the CT head scans done in the emergency department during the study period were included. The results showed that CT head was the most commonly prescribed CT scan in the ED. Among these 66.29% of scans were normal while 33.7% scan had positive findings. Thus almost two third of the total CT head scans done in the emergency department were normal. This shows a misuse of the emergency CT services. The reasons being high volume of patients, inadequate staffing, time constraints leading to lack of detailed history taking and physical examination, availability of free CT services, uncertainty on clinical diagnosis in most instances on part of some ED physicians; important factor being lack of awareness/implementation of internationally standardized guidelines as a reference before ordering CT scan.^10^

As a result of this study radiology department LRH devised and implemented radiology request forms for quality assurance for selection of both adults and children for CT scan head presenting to ED in order to ensure judicious and rational use of CT services in an emergency setting.

## CONCLUSION

CT head overuse is common especially in an emergency setting. Emergency physicians should be encouraged to perform a detailed history & physical examination and exercise overall judicious rational use of CT scan especially in pediatric CT head keeping the international guidelines as a standard protocol for patient selection.

### Author`s Contribution

**TN** conceived and final approval of article.

**TN, TA and NN** did manuscript writing.

**TA, NN and FM** did data collection.

**TA and FM** prepared the results.

**NN** did manuscript editing, review and final approval of manuscript.
